# 3D-Bioprinted Multifunctional Nanocomposite Scaffolds for Alveolar Bone–Periodontal Ligament–Root Cementum Regeneration: A Narrative Review

**DOI:** 10.3390/biomimetics11060425

**Published:** 2026-06-15

**Authors:** Angeliki Tsantiri, Nikolaos I. Mourkiotis, Hector Katifelis, Xanthippi Dereka, Maria Gazouli, Nefeli Lagopati

**Affiliations:** 1 Laboratory of Biology, Department of Basic Medical Sciences, Medical School, National and Kapodistrian University of Athens, 11527 Athens, Greeceklausmurk11@gmail.com (N.I.M.); mgazouli@med.uoa.gr (M.G.); 2 Department of Periodontology, School of Dentistry, National and Kapodistrian University of Athens, 11527 Athens, Greece; 3 School of Science and Technology, Hellenic Open University, 26335 Patra, Greece; 4 Biomedical Research Foundation, Academy of Athens, 11527 Athens, Greece

**Keywords:** 3D bioprinting, scaffolds, nanotechnology, nanocomposites, multifunctionality, periodontal regeneration

## Abstract

Periodontal disease remains one of the leading causes of tooth loss worldwide, highlighting the need for effective regeneration of alveolar bone, periodontal ligament, and cementum. The structural complexity and unique biological behavior of these tissues have historically posed significant challenges for clinical regeneration strategies. The primary therapeutic approach used is guided bone regeneration; however, it has certain limitations, such as morbidity, low structural integrity and dimensional stability. Recent advances in 3-dimensional (3D) bioprinting have made it possible to fabricate customized scaffolds with precise architecture and spatial organization that closely mimic normal periodontal structures. The incorporation of multifunctional nanocomposite biomaterials and nanoparticles further enhances the performance of the scaffolds by increasing mechanical strength, bioactivity and controlling degradation rates. These advanced scaffolds function as dynamic microenvironments that support cell adhesion, proliferation and differentiation, ultimately promoting tissue regeneration. Furthermore, their multifunctional properties allow for the controlled release of growth factors, anti-inflammatory and antimicrobial agents, as well as the incorporation of stem cells and bioactive molecules that facilitate angiogenesis. This review investigates and critically evaluates modern approaches for the regeneration of periodontal tissues through scaffolds, biomaterials and 3D bioprinting technologies, as well as to assess their effectiveness compared to established clinical practices.

## 1. Introduction

The periodontium is a complex system of tissues, comprising alveolar bone (AB), periodontal ligament (PDL), cementum (CM) and gingiva, which work in a coordinated manner to stabilize the tooth and maintain masticatory function. In periodontitis, microbial biofilm accumulation triggers a chronic inflammatory response that progressively destroys the periodontal tissues and is a main cause of tooth loss worldwide [1]. The primary goal of periodontal regeneration is to restore the damaged tooth-supporting structures through the formation of new AB, CM, and properly oriented PDL fibers. In this context, regenerative strategies aim to re-establish both the structure and the functional integrity of periodontal tissues [2]. The significance of periodontal regeneration extends beyond tooth preservation; it represents a key element in maintaining overall oral and systemic health. Periodontitis-induced tissue loss compromises both function and aesthetics, affecting mastication, phonation, and quality of life. Moreover, periodontitis has been linked to systemic inflammatory disorders, including cardiovascular disease, rheumatoid arthritis, metabolic syndrome, chronic kidney disease and diabetes mellitus [3,4,5].

The tissues affected by periodontitis have a limited intrinsic regenerative capacity due to disrupted vascularization, compromised cellular niches, and the absence of a coordinated signaling environment [3]. Conventional therapies such as scaling and root planing can reduce bacterial load and inflammation; however, they are often unable to fully restore the complex architecture of lost periodontal tissues, particularly in extensive lesions [2]. More specifically, scaling and root planing commonly result in the formation of a long junctional epithelium rather than true periodontal regeneration [1]. In this type of attachment, the root surface is attached via hemidesmosomes, which provide less protection to the periodontium than the connective tissue fibers embedded into the CM. As a result, patients who do not adequately control dental biofilm accumulation or who present with impaired immune responses may remain susceptible to recurrent periodontitis. To date, guided tissue regeneration (GTR) is considered as the gold-standard treatment for periodontal defects involving the AB, PDL and CM [6]. Over the past decades, numerous studies have highlighted the importance of GTR and contributed to the development of modern periodontal regenerative approaches. These studies established the fundamental principles of GTR, including wound stabilization, space maintenance, the recruitment of appropriate cells and cell differentiation [2,7]. Despite its clinical and biological success, the results of GTR remain variable, as they depend on the defect morphology, patient factors, and surgical technique and materials. The unpredictability of the regenerative outcome may be associated with the presence of oral bacteria and the unvascularized root surfaces within the periodontal environment [7]. Complications such as root resorption and ankylosis have also been reported. Furthermore, chronic periodontitis may reduce the regenerative capacity of the PDL cells, affecting overall tissue regeneration [8,9]. Since the periodontium consists of structurally and functionally distinct yet highly interconnected tissues [1,7], complete and functional regeneration continues to be challenging [2]. Consequently, healing often results in the formation of scar-like tissue rather than the full restoration of native periodontal architecture, as described by Vaquette et al. [7].

Epidemiological studies show that >50% of adults worldwide are affected by periodontal disease, and its occurrence increases with age [4]. Conventional regenerative approaches focus on individual tissues without fully restoring the biological interface that provides natural tooth adhesion and mechanical stability [1,3]. These limitations have led to the exploration of modern developments that will enable the complete regeneration of periodontal tissues. Continuous progress in biomaterials, nanotechnology and tissue engineering has made this vision more realistic [10]. Current regenerative therapies have significantly improved periodontal treatment outcomes; however, predictable reconstruction of the entire periodontal complex has yet to be achieved. Multifunctional bioprinted scaffolds have emerged as a promising alternative because they can combine tissue-specific architecture, controlled bioactive factor delivery, and spatially guided regeneration within a single construct [10,11,12].

Biomaterials and nanotechnology are increasingly recognized as major contributors to progress in regenerative medicine, mainly because they offer better control over the biological and mechanical processes involved in tissue healing. Unlike conventional biomaterials, modern nano-reinforced scaffolds do not simply serve as passive structural supports. Instead, they actively interact with cells and biological signaling pathways, influencing tissue regeneration at both the cellular and molecular levels [11]. The convergence of nanotechnology and biomaterials is particularly significant in tissue engineering (TE), where advanced scaffold systems are being developed for the restoration of complex tissues [12]. These scaffolds are manufactured ex vivo, using 3D printing technologies, and are implanted to regenerate damaged tissue. To achieve successful periodontal regeneration, scaffolds should support cell adhesion, proliferation, and differentiation, while also providing appropriate biological cues and maintaining adequate space for the regeneration of AB, PDL and CM [10].

Nanotechnology has significantly advanced tissue engineering by enabling interventions at the nanoscale, where most biological interactions naturally occur. Nanostructures such as nanoparticles, nanofibers, and nanocomposite hydrogels increase the bioactivity of scaffolds by improving protein adsorption and enabling the controlled release of bioactive molecules [11,12].

At the same time, the combination of nanotechnology and tissue engineering has led to the development of scaffolds that mimic the microenvironment of the extracellular matrix (ECM) more effectively. This improves material-cell interaction and allows for a more controlled delivery of bioactive signals and growth factors [12]. Periodontal regeneration is a particularly demanding process since each tissue exhibits distinct structural and biological characteristics, despite its close functional interdependence. Consequently, monophasic scaffold approaches are often insufficient for achieving organized and functional regeneration. This limitation has led to the development of multiphasic scaffolds, which consist of distinct compartments with different architectural and biochemical properties. These scaffold systems are designed to better reproduce the hierarchical organization of the periodontium and to address the complex interfaces between AB-PDL and PDL-CM [7]. Overall, the contribution of biomaterials and nanotechnology lies in their ability to bridge the gap between biology and engineering, transforming regenerative techniques into predictable, patient-specific, and functionally integrated therapies, bringing clinical translation closer to reality [7,10].

Unlike many previous reviews that discuss periodontal regeneration, biomaterials, or 3D bioprinting separately, the present review focuses specifically on multifunctional nanocomposite scaffolds developed for the coordinated regeneration of the entire periodontal structures, including AB, PDL, and CM. While a considerable number of studies have explored the regeneration of individual periodontal tissues, relatively few have critically examined strategies aimed at achieving simultaneous and functional regeneration of all three highly specialized and interdependent periodontal components within a single regenerative platform. In this context, the present review emphasizes the emerging potential of multiphasic and bioprinted scaffold systems designed to better reproduce the hierarchical organization and biological complexity of the native periodontium.

This review aims to summarize and critically evaluate how the latest advances in 3D-bioprinted multifunctional nanocomposite scaffolds can contribute to the comprehensive regeneration of the periodontal unit, combining principles of industrial design and biological signaling. Additionally, potential challenges and future perspectives associated with the clinical application of these regenerative approaches are highlighted.

## 2. Materials and Methods

A search was conducted in the international scientific literature, using relevant search terms both individually and in combination with one another, including “nanotechnology”, “nanomaterials”, “3D scaffolds”, “3D bioprinting technologies”, “tissue engineering”, “periodontal regeneration”, and “alveolar bone-periodontal ligament–cementum regeneration”. The main databases used were PubMed, ScienceDirect, and Google Scholar. For this narrative review, articles published in English after 2014 were selected based on their relevance. Initially, a screening was conducted based on the titles of the identified articles, and subsequently, a second screening stage was performed on the selected articles based on their abstracts. Additional studies were identified and included through a manual search of the bibliographic references of the articles already selected.

## 3. Three-Dimensional Bioprinting Technologies in Tissue Regeneration

3D printing is a technological process that enables the construction of solid objects layer by layer using computer-aided design (CAD) models. It is also known as additive manufacturing (AM) or rapid prototyping techniques and involves sophisticated, flexible and automated engineering methods that significantly simplify the manufacturing process. These technologies, due to the ever-increasing number of publications, are characterized as the third industrial revolution [13]. They find applications in the health sciences through 3D bioprinting, which is based on the principle that living tissues or entire organs can be created using mixtures of cells or biospheres via AM. For this purpose, “biοinks” are used, i.e., mixtures of living cells, biopolymers, and hydrogels that function as a temporary scaffold supporting cell viability and growth [14,15]. According to Papaioannou TG et al., the basic principles of bioprinting include biomimicry, self-assembly, and the use of microstructural building blocks. Biomimicry aims to replicate the natural microarchitecture and mechanical properties of tissues, while the nanostructured features of printed surfaces modulate cellular responses, including attachment, migration, and growth. At the same time, the 3D microenvironment plays a significant role in regulating cellular differentiation and tissue organization. Self-assembly is based on the ability of cells to organize autonomously when placed under suitable conditions, producing an extracellular matrix and communicating via signals that guide tissue structure and function [16]. Finally, the microtissue-based approach uses small and organized tissue units. When combined, these units promote the formation of functional tissue more effectively. They also improve cell viability and better mimic the natural tissue architecture and function. Successful tissue regeneration requires a balance between mechanical stability and biological activity [17].

### 3.1. Types of 3D Bioprinting

Depending on the mechanism of biomaterial deposition and the formation of the structure, 3D bioprinting can be divided into four main modalities, each of which has different operating principles and applications:

- Stereolithography/vat polymerization,
- Droplet-based bioprinting,
- Extrusion-based,
- Laser-assisted bioprinting [15,18,19,20].

**Figure 1:**
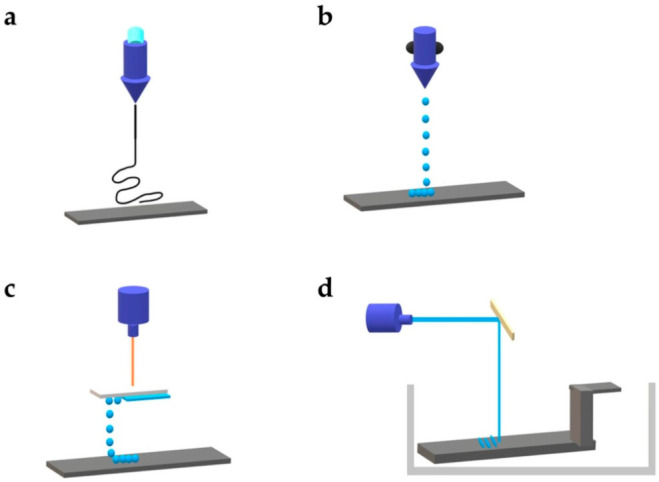
Schematic elucidating 3D Bioprinting modalities. (**a**) Extrusion bioprinting, (**b**) Droplet-Based bioprinting, (**c**) Laser-based bioprinting and (**d**) Vat polymerization technique.

These 3D bioprinting approaches are illustrated in Figure 1.

#### 3.1.1. Vat Polymerization/Stereolithography

Stereolithography (SLA) was the first commercial 3D printing technique, introduced in 1984, and is based on vat photopolymerization [21]. The method involves curing liquid resin layer by layer through controlled exposure to a laser (ultraviolet or visible spectrum), creating 3D objects designed on a computer. Subsequently, variations such as digital light processing (DLP) and continuous DLP (cDLP) were developed, which allow simultaneous curing of the entire layer using a system of microscopic mirrors and faster printing, as well as the two-photon polymerization (2PP) technique, which offers the construction of 3D structures at the nanoscale with high precision [15,21]. In bioprinting, SLA is used for the construction of complex scaffolds with cells or biomaterials, utilizing photosensitive bioinks. It is characterized by high resolution, fast printing speed, regardless of structural complexity, and the absence of a nozzle, which prevents clogging issues. The materials used include polymers such as PEG (Polyethylene glycol) and PCL (Polycaprolactone), as well as composite materials, ceramics, or titanium, depending on the application [17,21]. Nevertheless, this technique has limited applicability for the bioprinting of living cells because only a few suitable biocompatible photopolymers are currently available [19].

#### 3.1.2. Droplet-Based Bioprinting

In 1988, Klebe introduced the concept of “cytography,” which formed the basis for droplet-based bioprinting techniques. These mainly include electrohydrodynamic jetting and inkjet printing, while acoustic printing and microvalve bioprinting are used less frequently [17,22]. Electrohydrodynamic (EHD) jetting is based on the creation of a strong electric field that causes the ejection of droplets from a thin liquid stream, offering high resolution but requiring careful control due to potential effects on cells [23]. Inkjet bioprinting is the most established form and is distinguished into continuous inkjet printing (CIJ), where the biomaterial is continuously ejected through a nozzle and drop-on-demand (DOD) printing, with the latter providing greater precision and control over the position and ejection of the droplets. The DOD technique relies on different types of nozzles, which are activated by short electrical pulses and classified into piezoelectric, thermal, or electrostatic systems, allowing controlled droplet ejection [13,21,23]. In the inkjet bioprinting technique, and more specifically in the DOD method, high cell viability (~80%) is achieved and it is widely used for printing bone, cartilage, and skin. In contrast, it is important to use low-viscosity bioinks (3–12 MPa/s) to avoid nozzle clogging [22].

#### 3.1.3. Extrusion Bioprinting

Extrusion-based 3D bioprinters, also known as bioplotting, direct ink writing, or additive manufacturing (AM), represent the most widely used bioprinting technology due to their low cost, ease of use, and reliability in fabricating 3D tissue scaffolds with complex architectures using various biomaterials [24,25]. In this method, the biomaterial is continuously extruded through a syringe nozzle. The extrusion process is driven either by pneumatic systems using compressed air or by mechanical systems, such as piston- or screw-based mechanisms, enabling the layer-by-layer fabrication of 3D structures [17,22]. Piston extrusion systems offer better flow control, especially for high-viscosity materials, while screw extrusion ensures even greater precision and resolution. Nonetheless, there is a risk of cell damage within the biomaterial from the screw, and it presents cleaning difficulties. The use of materials with pseudoplastic properties and the reduction in printing speed can significantly limit this cell damage during the process [17,26]. There is also a newer variation in this technique, “cryobioprinting”, which aims to optimize cell viability and create more stable scaffolds. It prints directly at −40 °C and freezes the material in a controlled manner, forming scaffolds that show better attachment and survival of mesenchymal stem cells and more closely resemble the natural structure of tissue. It is mainly used in the construction of cartilage tissue, but it also has potential for bone and other soft tissues [26]. Extrusion bioprinting is the most widely used method in periodontal tissue engineering, as it allows the use of high-viscosity biomaterials that provide mechanical support to scaffolds and the incorporation of multiple cell types needed for the regeneration of hard and soft periodontal tissues [27].

#### 3.1.4. Laser-Based Bioprinting (LBB)

The laser-based bioprinting (LBB) technique is based on the principle of laser-induced forward transfer (LIFT) [22]. It utilizes biomaterials with high cell density and precise microscopic organization. Laser pulses activate an absorbing metal layer, generating a high-pressure bubble that propels the biomaterial onto the substrate at speeds of up to 5 kHz [28]. LBB is a nozzle-free and contact-free printing technique, which reduces the risk of clogging and enables the use of high-viscosity biomaterials, thereby enhancing the fabrication of complex tissue structures [22]. In addition, it provides high resolution (>20 μm), precision in orientation and spatial positioning of cells in 3D structures and prints at high cell density (~108 cells/mL), suggesting the suitability of the technique for applications in tissue engineering. It is worth noting, however, that the use of lasers can cause damage to cells, leave metal residues from the absorbent layer and have limited speed in large-volume production. The range of biomaterials that are directly compatible with the technique is also limited, so more studies and time are needed to make it fully functional [13,22]. Recent reviews emphasize that this technique faces difficulties in transitioning from the laboratory to clinical application, while improvements are needed in reproducibility, process reliability and production automation [26].

**Figure 2:**
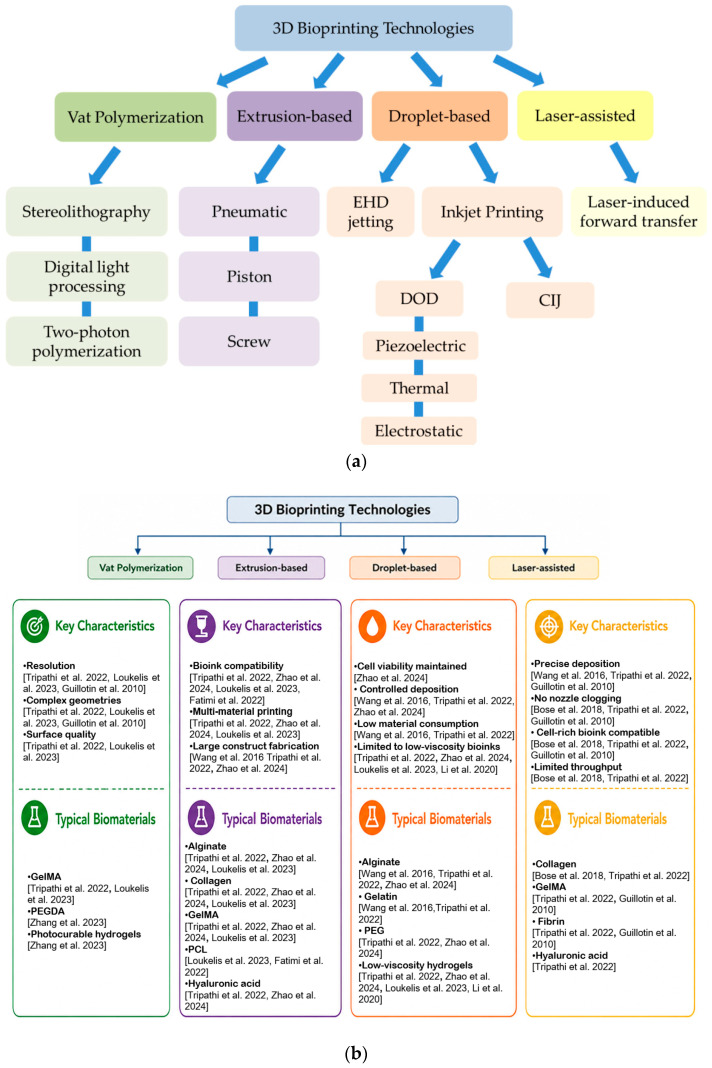
(**a**). Classification of 3D bioprinting methods for scaffold fabrication. (**b**). The major categories, key characteristics and commonly used biomaterials are summarized for each technique [13,14,17,18,20,21,23,28,29].

Figure 2a,b provides a summarized overview of the above categories and how they are classified.

Overall, the comparison presented in Table 1 highlights the distinct advantages and limitations of the major bioprinting techniques used in periodontal tissue engineering. Extrusion bioprinting remains the most widely applied approach because of its cost-effectiveness, mechanical stability, and compatibility with a broad range of biomaterials [13,17,18,27]. Current research is increasingly focused on hybrid bioprinting strategies aimed at more accurately reproducing the hierarchical organization of periodontal tissues [18,26,29].

### 3.2. Types of Bioinks

Bioink is the main component used in 3D bioprinting and can be classified into two main categories. The first category includes scaffold-free bioinks, which do not contain a supporting material for cell stabilization and therefore consist almost entirely of cells, such as tissue spheroids, cell pellets, and tissue strands. The second category includes scaffold-based bioinks, which combine cells with carrier materials that support cell attachment and growth. These bioinks commonly contain decellularized extracellular matrix (dECM), hydrogels, microcarriers, or concentrated cell suspensions.

**Figure 3:**
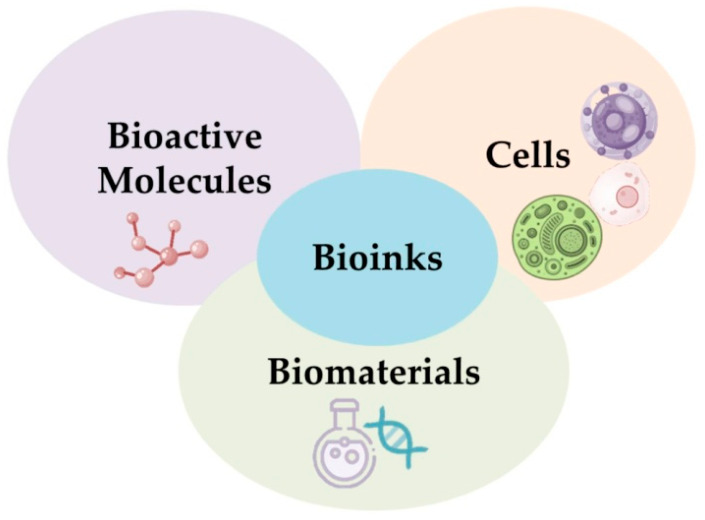
The main components of the commonly used bioinks; cells, bioactive molecules and biomaterials.

Depending on the intended application, bioinks are typically composed of cells, biomaterials, and bioactive molecules, as illustrated in Figure 3. Bioinks containing multiple biomaterials often demonstrate improved bioprinting performance compared to single-material systems because they combine the advantageous properties of different biomaterials. In scaffold-based systems, scaffolds provide structural support for cell growth and tissue formation while maintaining a favorable microenvironment for cell viability [30,31]. The biomaterials used in bioinks may be natural, synthetic, or hybrid combinations of both [32]. An ideal bioink should possess suitable physicochemical properties, including good printability, shape fidelity after deposition, and cytocompatibility during the crosslinking process. Furthermore, it should facilitate nutrient transport and support long-term cell viability within the printed construct [31].

#### 3.2.1. Natural Bioinks

Natural biomaterials include gelatin, collagen, alginate, hyaluronic acid, cellulose, chitosan, chitin and dECM. They accurately mimic the tissue microenvironment of ECM and offer high cell viability, while most of them contain RGD sequences (Arginine-Glycine-Aspartate (Arg-Gly-Asp)) for cell attachment. They are characterized by good biological behavior as they exhibit bioactivity, high biocompatibility and controlled biodegradability, while chitosan is also utilized for its antimicrobial properties. Despite these advances, their use in scaffolds is limited due to low mechanical strength, so they often need to be incorporated as hybrid or reinforced materials with synthetic materials to optimize the properties of the final material [2,32,33].

#### 3.2.2. Synthetic Bioinks

Synthetic biomaterials are divided into three main categories: synthetic polymers, bioceramics, and metals. Synthetic polymers mainly include aliphatic polyesters such as PCL, PLA (Polylactic Acid), PGA (Polyglycolic Acid), PLGA (Poly(lactic-co-glycolic) acid), and PEG, the latter of which is used as a controlled drug release material. These polymers have good mechanical strength, the ability for printing processing into complex shapes and low bioactivity, which often requires reinforcement with other materials. Another characteristic of aliphatic polyesters is that they exhibit a slower degradation rate compared to natural polymers and bioceramics [2]. Bioceramics are inorganic synthetic materials with the main forms being Hydroxyapatite (HA) [34,35]—crystalline or amorphous, β-Tricalcium Phosphate (β-TCP), α-Tricalcium Phosphate (α-TCP), BCP (Biphasic Calcium Phosphate)—a mixture of HA + TCP, TTCP (Tetracalcium Phosphate), DCP/Brushite (Dicalcium Phosphate), MCPM (Monocalcium Phosphate Monohydrate), 45S5 bioglass and other CaP with different Ca:P ratios. Bioceramics exhibit: high osteoconductivity, osteoinductive capability, biocompatibility, difficulty in processing due to very high temperatures, and due to their brittle nature, they are preferred as reinforcements in composite materials [2,36]. Bioceramics can also improve CM formation, but silicon-based bioceramics appear to promote CM formation to a greater extent [8]. In the category of metals belong Titanium (Ti), titanium alloys (Ti-alloys), Magnesium (Mg) and magnesium alloys, which are characterized by high mechanical strength, hardness, and biocompatibility (mainly Ti) and complete degradation (in the case of Mg), while exhibiting limited bioactivity and difficulty of removal (titanium, non-degradable) [2,14].

While bioinks provide the biological and structural framework required for 3D bioprinting, their performance is often limited by insufficient mechanical properties, bioactivity, or tissue-specific functionality. To overcome these limitations, nanomaterials are increasingly incorporated into bioinks and scaffold systems to enhance their physicochemical characteristics and biological performance. Consequently, nanocomposite biomaterials have emerged as a promising strategy for improving periodontal tissue regeneration [2,20,32,33,37].

## 4. Nanocomposite Biomaterials on Scaffolds for Periodontal Regeneration

### 4.1. Nanoparticulate Composites

Νanocomposite is a material with more than two phases, at least one of which is at the nanoscale, and its dimensions range roughly from 1 to 100 nanometers [37]. In nanocomposites, the nanostructure acts as a reinforcing element within a matrix. Combining two or more different biomaterials produces a “synergistic effect” with the overall properties of the merged materials and improved mechanical, biological, and kinetic properties. Therefore, scaffolds made of such materials are referred to as “composite” or “hybrid,” and when three biomaterials are incorporated, they are referred to as “trimeric” [2]. The addition of nanoparticles to bioink offers regulatory and biological enhancement due to the shape, size, concentration and surface chemistry, leading to improved design of bioactive tissue engineering scaffolds [20]. Nanocomposite materials are divided into three major categories based on the main matrix material: Polymer Matrix Nanocomposites (PMNC), Ceramic Matrix Nanocomposites (CMNC) and Metal Matrix Nanocomposites (MMNC). Within this group, the first category is the most widespread, with a multitude of applications in 3D printing and is preferred due to low cost, ease of processing, flexibility, and high durability [38].

### 4.2. Role of Nanomaterials in Scaffold Enhancement

The physicochemical and biological properties of nanomaterials differ significantly from those of their bulk counterparts because their small size results in a higher surface-to-volume ratio and enhanced cellular interactions. Nanoparticles can release ions or bioactive molecules that activate signaling pathways, influencing cellular behavior. Additionally, they offer favorable biocompatibility, antibacterial properties and the ability to promote regeneration [39,40].

The structure of the ECM includes nanoscale elements, so nanosynthetic scaffolds exhibit an architecture much closer to the natural ECM compared to other scaffolds. Scaffolds with nanoscale porosity and surface characteristics similar to those of the natural ECM create a microenvironment that enhances cellular processes. Furthermore, nanoparticles possess a large surface area and high charge density, allowing stronger interactions with negatively charged bacterial surfaces. As a result, nanoparticles have attracted considerable interest in dental applications because of their broad-spectrum antibacterial activity. Silver nanoparticles show intense antibacterial and anti-inflammatory effects, promoting healing, while Ag-containing composite scaffolds have also been shown to enhance AB regeneration. Additionally, ZnO nanoparticles have dual action as they limit the formation of microbial biofilms while simultaneously contributing to osteogenesis [40].

The distribution and concentration of nanoparticles within the matrix material strongly affect the properties of 3D printed objects. Homogeneous distribution contributes to the enhancement of mechanical strength, which is achieved through methods such as sonication, ultrasonic processing or mixing with the aid of surfactants. In contrast, the agglomeration of nanoparticles weakens mechanical strength, so it is critical to avoid the formation of aggregates throughout the printing process [41]. A high concentration of nanoparticles in the matrix causes significant improvements in the thermal and electrical properties of the printed scaffolds, but excessive loading leads to agglomeration and consequently to a degradation of mechanical properties. On the other hand, at low concentrations of nanoparticles, only small changes occur in the material properties, thus highlighting the importance of the proper selection of concentration and distribution of nanoparticles. Equally important are the interfacial interactions developed between nanoparticles or microspheres and the polymer matrix, as these determine mechanical strength, biocompatibility and the ability for controlled drug release. In cases where these interactions are insufficient, adhesion decreases, and the material’s performance is degraded [42].

3DP can accurately control the dispersion of nanoparticles, a factor that affects the release profile and targeted delivery. This makes it possible to design personalized drug delivery systems that respond to specific biological stimuli (pH, temperature) and can release the drug in a controlled manner. The incorporation of nanoparticles, such as carbon nanotubes, graphene, HA or silicon dioxide, into matrices enhances mechanical strength, stiffness, degradation rate, as well as surface properties, so that they approach those of natural tissues [43].

The regenerative benefits of nanomaterial-enhanced scaffolds ultimately depend on their ability to meet the distinct biological and structural requirements of individual periodontal tissues. Since alveolar bone, periodontal ligament, and cementum exhibit different compositions, architectures, and functional properties, the selection and design of nanomaterials must be tailored to the specific regenerative objectives of each tissue compartment [8,20,39,40].

### 4.3. Nanomaterials Used in Periodontal Regeneration Scaffolds

#### 4.3.1. Nanomaterials for Bone Reinforcement and Osteogenic Signaling

Biomaterials employed in regenerative bone engineering should possess biocompatibility, osteoconductive properties, and osteoinductive potential. In addition, the re-establishment of local vascularization at the defect site is critical for successful healing and tissue remodeling [44]. HA is the most commonly used naturally nanostructured material for AB regeneration. It is based on calcium phosphate, and its chemical composition and crystal structure resemble the natural mineral components of bone. When HA is incorporated into the defect, osteoblasts attach to it and produce osteoid, which is then converted into mature bone, without any fibrous tissue interfering. However, inorganic elements such as HA have low mechanical strength, so they are combined with organic elements such as collagen, gelatin and chitosan [45]. HA and calcium phosphate are often incorporated into 3D-printed bone scaffolds due to their excellent biocompatibility, bioabsorbability, and ability to promote osteogenic activity and bone tissue formation while supporting stem cell function [11,27,36]. Studies using hydroxyapatite-gelatin nanocomposites showed cell compatibility and proliferation of dental mesenchymal stem cells, paving the way for successful bone healing and AB regeneration [45]. Equally frequently used is TCP, which has two forms, α-TCP and β-TCP, with β-TCP being more commonly used due to better bioactivity and degradation rate. HA and TCP can coexist in different proportions within composite scaffolds and can be enhanced with trace elements such as magnesium or strontium, allowing greater mechanical strength compared to pure TCP scaffolds, faster osteogenesis and angiogenesis as shown by animal studies [44].

In addition, biodegradable biopolymers such as PLGA and PCL are used as nanoparticle carriers and can incorporate therapeutic agents such as antibiotics and growth factors, providing controlled and sustained release that contributes to the process of osteogenesis. PLGA does not have satisfactory mechanical strength on its own, so it needs to be reinforced with ceramic nanomaterials such as nano-hydroxyapatite (nHA), PCL, and fluorohydroxyapatite. PCL, on the other hand, has a slow biodegradation rate, so it cannot be used as a scaffold material on its own [11]. PCL is also a durable material with mechanical strength suitable for bone regeneration applications. PCL can be combined with various polymers and inorganic materials and because it exhibits a low glass transition temperature, processing in 3D printing is facilitated [27].

Researchers constructed a polymer scaffold (PLGA/PCL) with nanofibers, which was then modified by adding silver nanoparticles (Ag), polydopamine (pDA) nanoparticles and a collagen coating to improve biocompatibility, osteogenesis, and antimicrobial activity. In vitro cell culture experiments exhibited improved cell–surface interactions, proliferative activity and increased expression of osteogenesis markers, while mouse models showed significant bone regeneration (~31.8%) [46].

Gold nanoparticles (AuNPs) also play an important role in the proliferation and differentiation of osteoblasts and mesenchymal cells. AuNPs are mainly used in imaging techniques for monitoring and for their antibacterial action, factors that make them promising tools [11,40].

A key growth factor that is particularly important in bone regeneration is bone morphogenetic protein 2 (BMP-2). It is usually loaded onto nanomolecular carriers that release it in the target area with control over time and quantity, offering more targeted and regulated support for osteogenesis [11,44]. Studies have shown that BMP-2 dramatically increases bone height in animals, but its administration requires caution as it can cause excessive bone growth, fat tissue formation and poor-quality bones [11]. Another nanomaterial that can be used for bone regeneration is bioactive glass (BG), which is based on silicon dioxide, is amorphous and creates an apatite-like structure that resembles bone. Laponite is also a silicon-based nanomaterial that releases magnesium, lithium, and silicic acid ions during degradation. These ions promote osteogenesis and angiogenesis. This effect was confirmed in an experimental study in which laponite was combined with GelMA (gelatin methacrylate). The resulting hydrogel demonstrated greater stability, durability, and cytocompatibility compared to pure GelMA. Carbon nanotubes (CNTs) and carbon nanofibers (CNFs) exhibit electrical conductivity, large surface area and high mechanical strength, while their geometry contributes to their better integration [20]. The nanocomposite materials used for AB regeneration also need to carry angiogenic factors because, without a blood supply, osteogenesis cannot occur. One such application is VEGF-modified black phosphorus nanosheets in DNA (deoxyribonucleic acid) hydrogels embedded in PCL scaffolds, where AB and vascular network formation were observed [29]. Another example is the addition of calcium silicate nanoparticles surface-modified with polydopamine (PDACS) in PCL scaffolds, which indicated the same fact [20].

CeO_2_ nanoparticles exhibit antioxidant, anti-inflammatory, antibacterial activity and angiogenic potential. At the same time, they promote osteogenic commitment of human PDL stem cells (hPDLSCs) by enhancing ALP (alkaline phosphatase) efficiency and the production of mineralized nodules. This finding is also supported by the study of Sh. Ren et al. observed that the combination of CeO_2_ nanoparticles with porous glass scaffolds or gelatin-alginate scaffolds promotes both new AB formation and the proliferation of stem cells. Nevertheless, the exact mechanism by which CeO_2_ NPs affect the differentiation of hPDLSCs has not yet been fully clarified and requires further investigation [47].

There are also FeHA particles in this category, which exhibit superparamagnetic properties. These particles consist of nanocrystalline apatites in which iron ions (Fe^2+^/Fe^3+^) are incorporated in place of calcium ions. The magnetic signals generated by these nanoparticles can activate the hybrid scaffold layers in which they are incorporated, thereby enhancing cell proliferation and promoting the osteogenic properties of apatite-based hybrid materials. Unlike conventional superparamagnetic metal oxides, which may present cytotoxicity risks, FeHA represents a biocompatible, bioactive, and biodegradable magnetic phase. Studies applying external magnetic stimulation to magnetic scaffolds containing iron nanoparticles demonstrated enhanced osteoblast differentiation in vitro and increased new AB formation in vivo [44,48].

Mesoporous nanomaterials based on silicon oxide have also attracted interest, as research has shown AB regeneration in 3D scaffolds made of mesoporous silica fibers and gelatin. The use of these materials promotes the viability of osteoblastoid cells and the activity of alkaline phosphatase, the expression of genes that express collagen and other osteogenic molecules. Gelatin mimics the structure of extracellular matrix proteins, and the nanofibrous layers of silica provide contact points and guidance for cell spreading [49].

Ions (Na^+^, Mg^2+^, Si(OH)_4_, Li^+^) can be incorporated into the structure of nanoparticles or hydrogels and contribute to osteogenic effects through signaling pathways [40].

#### 4.3.2. Nanoparticles for PDL Regeneration Enhancement

The use of nanomaterials makes it possible to better replicate the native structure and nanofibrous architecture of PDL. Type I collagen, the main component of the extracellular matrix, is commonly used to produce nanofibrous scaffolds that support cell alignment and viability [50]. Similar findings were reported by Tian et al., who developed bioscaffolds composed of HA nanoparticles, hydrogel, and PDL stem cells. Their study demonstrated improved mechanical stability and enhanced organization of PDL-like tissue [51].

An additional study conducted by Zhu et al. showed that the application of GelMA hydrogel embedded with PDL stem cells enhanced the bioactivity of the cells. This was followed by the study of Yang et al., who aimed to further improve GelMA hydrogel by incorporating extracellular matrix components derived from porcine dental follicles to promote stem cell differentiation. The results demonstrated improved mechanical properties, as well as enhanced fibrogenesis and osteogenesis [52]. In the construction of the PDL the main challenge is the organization and the directed arrangement exhibited by the fibers of the PDL. The difficulty lies in producing soft tissue that is integrated between two metallized surfaces while simultaneously enduring various forces. A nanofiber-forming collagen fabrication method with high orientation is electrospinning. In cases where this technique is combined with bioprinting, it creates well-directed fibers that mimic the natural PDL [11]. The fabrication of collagen nanomembranes through neutralization and crosslinking using BDDGE produced scaffolds with 40 nm pores, closely resembling the structure of natural collagen fibers. These findings demonstrated that nanomorphology plays a critical role in the way cells perceive and interact with the scaffold [48]. Controlled cooling techniques can also promote the natural guidance of PDL fibers toward anatomically appropriate orientations. In addition, they enable the formation of elongated pores ranging from 3 to 20 μm with nanoscale organization, contributing to the reconstruction of the oblique and vertical PDL fiber bundles. At the same time, microgrooves generated through 3D bioprinting create substrates that guide cellular orientation, as cellular alignment was maintained for up to 21 days. In another study, 3D nanofibrous scaffolds fabricated using electrospun membranes combined PCL/PEG nanofibers with chitosan. These scaffolds guided cells toward vertical or oblique orientation in vivo, demonstrating that nanostructured systems mimic the functional organization of native PDL more effectively than two-dimensional membranes [15]. Another approach involved the fabrication of collagen-based fibrous scaffolds with a wavy architecture designed to better withstand the shear forces generated during mastication.

The FRESH technique allows this wavy morphology, which is characteristic of the natural waviness of PDL fibers and offers high cell viability and enhanced expression of proteins such as Cyclin D, E-cadherin and periostin under dynamic loading, demonstrating that geometry at the micro- and nanoscale translates into functional improvement of the regenerative process [53]. In in vitro experiments conducted by Xiaomin Lan et al., an electrospun scaffold made of PCL, collagen, and cellulose acetate was constructed, suitable for the survival and migration of PDLSCs. Simultaneously, ZIF-8 nanoparticles loaded with curcumin were incorporated into a collagen hydrogel for more controlled release of the anti-inflammatory molecule at the injury site. The scaffold-hydrogel with PDLSCs and curcumin-loaded ZIF-8 nanoparticles was used in injured PDL fiber models, creating a promising system for PDL regeneration with anti-inflammatory and healing effects [54].

#### 4.3.3. Nanoparticles for Cementogenesis Enhancement

The CM is a calcified connective tissue in which PDL fibers attach and insert, making the regeneration of its precise morphology particularly challenging [11]. Several studies have proposed materials such as nanoHA, nano-calcium phosphates, and bioactive polymers as promising systems for CM regeneration under favorable conditions. Although these materials have shown promising regenerative potential under favorable conditions, their beneficial effects are mainly related to the improvement of the local microenvironment and the reduction in inflammation, while complete CM regeneration has not been consistently demonstrated in experimental studies [55].

The regeneration of CM on the dental root surface represents a fundamental and demanding stage as it leads to the creation of new attachment through the anchoring of Sharpey fibers. Until recently, biomaterials and 3D biopolymeric PLGA scaffolds were mainly used as delivery platforms for cementoblasts and bioactive molecules with cementogenic activity, such as PDGF-BB (platelet-derived growth factor-BB). In recent years, however, increasing attention has been given to materials with nanostructured surfaces, as they have been associated with the activation of important signaling pathways, including Wnt/β-catenin, which plays a key role in both cementogenic and osteogenic differentiation [56]. At the same time, nanostructured hydrogels, such as fibrous hydrogels with controlled degradation, have been used as matrices to support the formation of inorganic layers on the root surface and the insertion of Sharpey fibers. However, complete functional regeneration remains challenging because of the difficulty in achieving precise spatial fiber orientation. In addition, combined strategies using 3D-printed scaffolds and PLGA nanoparticles for the controlled release of growth factors have shown that successful CM and periodontal tissue regeneration depends on the coordinated interaction between nanoscale cues, scaffold architecture, and biological signaling pathways [15].

An attempt was made by Sprio and collaborators to simulate CM by constructing fibrous scaffolds from FeHA nanoparticles coupled with cellulose acetate (CA). The FeHA/CA scaffolds produced through the electrospinning technique exhibited similar physicochemical and biological characteristics to natural CM, while their structure showed a dense fibrous network with a rough surface, low apatite crystallinity and low porosity. Such a structure resembles the acellular extrinsic fiber CM, i.e., the part of the cement responsible for the attachment of PDL fibers [48].

Proteins related to CM, such as CEMP1 (Cementum Protein 1), CGF (Cementum-derived Growth Factor), and CAP (Cementum Attachment Protein), as well as stem cells, play a crucial role in regeneration as they lead to the formation of an ECM with characteristics of CM. In contrast, the newly formed CM that is created has a low fiber density and contains cells. The goal of the regenerative process is to create an acellular extrinsic fibrous CM, as this contributes more to adhesion. By leveraging nanomaterials, this limitation is addressed [57]. More specifically, HA bioceramic materials with micro–nano hybrid surfaces create a favorable microenvironment that supports cementoblastic activity and the formation of a functional attachment interface, while simultaneously activating critical signaling pathways, such as Wnt. It can be observed that these nanostructured materials manage to mimic key characteristics of natural CM and support the creation of functional cementoblastic activity; nevertheless, further in vivo documentation is still required [4].

## 5. Multifunctional Bioprinted Nanocomposite Scaffolds

### 5.1. Biomimetic and Multilayer Design

As regards the design and architecture of multilayer scaffolds, they must possess appropriate structural, mechanical, and biological properties to support the regeneration of each individual tissue and promote functional interactions between them. Such scaffolds must follow the hierarchical organization of the periodontium and form separate but integrated zones. The architecture must be designed to control porosity and permeability in each layer. These elements determine cell migration, angiogenesis and the uniform distribution of nutrients, with macropores 100–700 μm in diameter favoring vascular infiltration and smaller pores of approximately 40 μm exhibiting higher cell density [58,59]. Similarly, Vaquette et al. developed a highly macroporous scaffold with a mean pore diameter of 220 ± 141 μm and PCL fiber diameters of 15.5 ± 1.9 μm, supporting tissue infiltration, structural stability, and periodontal tissue formation in vivo [60]. Pores of 300 μm and above contribute to effective osteogenesis, while studies show that PDLSCs are successfully cultured and multiplied on scaffolds with porosity of 85% and above, with an average pore diameter of 162 μm, in a range of 116–515 μm [58,59]. Equally important design features of scaffolds for AB regeneration are pore size and overall porous structure. The overall porous structure of spongy human bone ranges from 30 to 90%, with experimental data suggesting 70% as optimal, as a highly porous structure can reduce mechanical stability [2]. At the same time, the mechanical properties of each tissue must allow proper damping of masticatory forces and appropriate mechanobiological signaling. In addition, tensile strength, stiffness, and elasticity should be adapted to the functional requirements of the native tissue. Of particular interest is the PDL, which is characterized by anisotropic elasticity and viscoelastic damping, allowing the transmission of forces while maintaining homeostasis [58]. Therefore, when designing ideal scaffolds, it is necessary to reproduce the natural elasticity of the PDL (between 50 and 150 MPa) while at the same time presenting a gradual transition to the more rigid areas of the CM and AB, where the elasticity will progressively increase up to 1 GPa, in order to facilitate the normal absorption of loads. The behavior of periodontal stem cells is influenced by the stiffness of the substrate, as this affects the organization of integrins and the tension of the cytoskeleton. Rigid substrates are favorable for the osteogenic differentiation of PDLSCs, as they lead to increased expression of osteogenic markers and greater mineral deposition compared to soft substrates [61].

In addition, the scaffolds must be made of biodegradable materials such as collagen, chitosan, and alginate, as well as other polymers such as PLA, PGA (Poly(glycolic acid)), and PLGA, so that there is additional space to incorporate the produced cells. Degradation occurs through hydrolysis, enzymes, and volumetric erosion, while in periodontal regeneration, the appropriate rate of biodegradation ranges from approximately 6–12 weeks for the first phase, which involves covering and stabilizing the defect. The second phase takes 6–12 months and involves the complete reorganization and functional maturation of the tissue [9,58]. Finally, it is essential to create aligned scaffolds around the cells in order to facilitate the imitation of natural tissue. Structural alignment is particularly important in PDL regeneration, as the tissue consists of highly aligned collagen fibers. This organization improves cellular behavior and more closely resembles the architecture of native tissue, creating a favorable microenvironment. Experimental data show that scaffolds with aligned morphology promote PDLSC differentiation, more structured formation of the PDL and more effective regeneration of the AB. In addition, it has been shown that when the fibers in electrospun or 3D-printed scaffolds follow a specific orientation, mechanical loading is transmitted more effectively to the cellular environment [58].

### 5.2. Patient-Specific Multifunctional Design

In recent years, advances in imaging techniques and computational tools have enabled the development of personalized scaffolds, using data from computed tomography and CAD design that accurately render the geometry of the defect [62]. Even so, not all bioprinting techniques are equally suitable for periodontal applications, as the successful regeneration of the periodontal complex requires a high degree of architectural precision and spatial control. Rapid prototyping techniques are considered the most suitable, as they offer controlled internal structure and high reproducibility, even in very complex constructions. Compartmentalization is also of particular importance, as it allows the functional separation of individual tissues and the spatiotemporal control of regeneration, reducing complications such as ankylosis. Based on this approach, triphasic scaffolds have already been developed, such as PCL/HA frameworks, which demonstrate successful multiphasic periodontal regeneration and controlled release of bioactive factors in vivo according to Asa’ad et al. [2].

The multifunctionality lies in the fact that these scaffolds combine many functions simultaneously. In this context, gene therapy constitutes a particularly promising strategy, as it allows the transfer of modified genetic material to periodontal tissues with the aim of enhancing the endogenous production of growth and differentiation factors [63]. The gene can be introduced either through viral vectors or through stem cells that are cultured and implanted into the defect, improving effectiveness and reducing risk for the patient. Experimental data presented by Jin Liu et al. show that factors such as PDGF can support the regeneration of AB, PDL, and CM [4]. For complete functional restoration, tissue regeneration alone is not enough. The vitality of the tissues and their interaction with the surrounding environment must also be preserved. For this reason, in extensive defects, the development of a functional vascular and nervous network through bioprinting is necessary to ensure proper integration and long-term functionality of the tissue. At the vascularization level, techniques have already been developed for creating small-diameter vascular channels, such as the coaxial system with alginate and carbon nanotubes developed by Dolati and colleagues, while the construction of capillary vessels continues to be challenging. Simultaneously, approaches such as the creation of channels by removing printed fibers from hydrogels have led to permeable scaffolds with endothelial lining, according to Bertassoni et al. Similarly, nerve tissue regeneration is critical due to the limited self-healing capacity of neurons. Bioprinting has enabled the development of nerve channels with Schwann cells and stem cells, as well as the printing of nerve and glial cells, while newer techniques, such as microstereolithography with polymers like PEG, further enhance restoration capabilities [64]. Despite these advances, the fabrication of fully functional neurovascular networks remains one of the major challenges in periodontal tissue engineering. While several approaches have successfully generated vascular channels and supported endothelial cell growth in vitro, the formation of stable capillary networks and their integration with host vasculature after implantation remain difficult to achieve. Similarly, neural regeneration is considerably more complex than structural tissue regeneration, as the restoration of sensory and functional innervation requires precise guidance of neural cells and long-term integration with surrounding tissues. Consequently, the development of scaffold systems capable of simultaneously supporting angiogenesis, neurogenesis, and periodontal tissue regeneration continues to represent an important area of ongoing research [58,63].

### 5.3. Multiphase Bioprinting and Layered Regeneration

The simultaneous regeneration of the periodontal complex is particularly demanding, as the simple combination of stem cells and scaffolds is not sufficient to restore its natural architecture. It is important to note that the native periodontium exhibits a layered, or layer-by-layer (LBL), structure [8]. For this reason, layered cell-based approaches have been developed to better mimic the natural organization of periodontal tissues, with each layer containing specific materials, cells, and bioactive agents. Studies with multilayered scaffolds have shown that the regeneration of CM, PDL and AB occurs in a largely coordinated manner, although osteogenic activity may be slightly ahead [65]. For example, in a large preclinical ovine model, biphasic scaffold constructs combined with Bm-MSC or PDLC sheets demonstrated an increase in bone fill from approximately 10% at 5 weeks to nearly 30% at 10 weeks, as assessed by micro-computed tomography analysis, highlighting their capacity to support coordinated periodontal regeneration in vivo [60]. Recent studies have developed a tissue-engineered complex with an LBL structure in which layered membranes with different degrees of metallization were placed [65]. The results showed rapid and complete periodontal restoration in animal models, with proper tissue formation and orientation, demonstrating their importance in relation to conventional techniques. Advances in CAD and 3D printing technologies have enabled the development of multiphase micro-scaffolds designed to support complete periodontal regeneration through the controlled spatiotemporal delivery of bioactive agents. These scaffolds consisted of PCL/HA in a 90:10 weight ratio and had three consecutive distinct layers, each corresponding to the CM, the PDL and the AB, respectively. Each layer administered recombinant human amelogenin, connective tissue growth factor (CTGF), and BMP-2, respectively. The findings showed tissue formation both in vitro and in vivo with characteristics similar to those found in normal tissue, such as collagen fibers of the PDL and well-organized mineralized tissue [66].

At the same time, triple-layered nanostructured scaffolds based on biomaterials were developed and combined with growth factors, antibodies and pharmaceutical substances to mimic the structure of periodontal tissues and enhance the attraction of host cells without the need for exogenous cell transplantation. More specifically, the scaffold consisted of chitin-PLGA and nanoactive glass-ceramic, enriched with CEMP1, PRP (Platelet-Rich Plasma), and FGF-2 (Fibroblast growth factor 2) for targeted regeneration of CM, AB and PDL. After implantation in animal periodontal defects, regeneration was achieved, demonstrating that even the cell-free scaffolds with appropriate bioactive signals can achieve complete periodontal regeneration through the activation of host cells [4]. Taken together, these findings suggest that multiphasic scaffold systems currently represent one of the most promising strategies for periodontal tissue engineering. Nevertheless, challenges related to vascularization, manufacturing reproducibility, and long-term clinical validation continue to hinder their translation into routine clinical practice.

**Figure 4:**
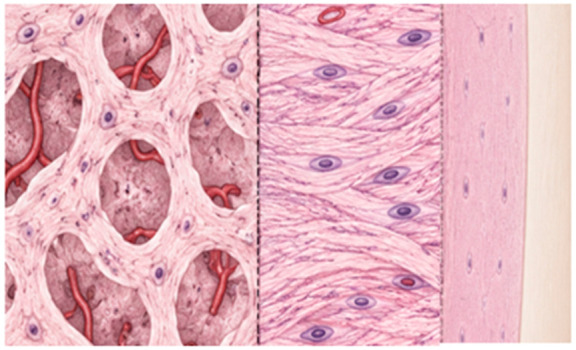
Schematic histological representation of the tissues involved in periodontal regeneration. From left to right: AB, PDL, CM and tooth dentin.

**Figure 5:**
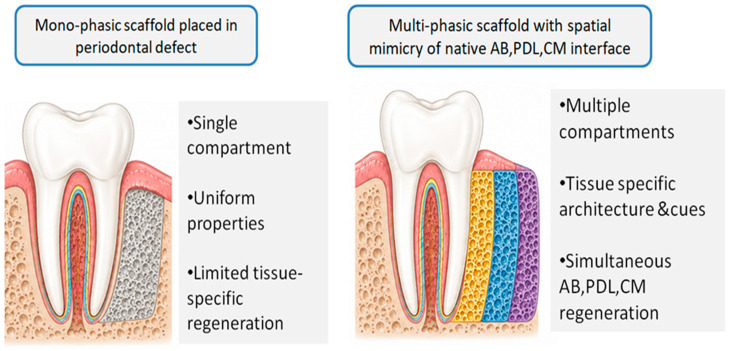
Schematic illustrations of the application of a 3D bioprinting scaffold in periodontal defects: (**Left image**): Ιncorporation of a monophasic scaffold. (**Right image**): Ιncorporation of a triphasic scaffold.

Figure 4 and Figure 5 schematically illustrate the structural organization of the periodontal complex and the hierarchical arrangement of the major periodontal tissues targeted during regenerative approaches.

To further summarize the different regenerative strategies and facilitate comparison among representative nanomaterial-based scaffold systems, Table 2 presents selected experimental studies, including scaffold composition, fabrication methods, biological components, regenerative outcomes, and key translational limitations.

### 5.4. Toxicity and Safety

Of particular importance in these scaffolds is the toxicity that may be caused by the use of nanomaterials in their construction. Once entering the body, nanomaterials interact directly with the biological environment and can lead to cellular and systemic toxicity to varying degrees. When nanoparticles come into contact with blood components, they can lead to hematological toxicity, while when they accumulate in organs such as the liver, spleen and kidneys, they can cause immune and endocrine disorders. The main mechanism of nanotoxicity is due to the overproduction of reactive oxygen species (ROS), which are responsible for oxidative damage to DNA, lipids, and proteins, causing serious long-term pathological consequences, such as carcinogenesis, neurodegenerative diseases and cardiovascular, renal, or pulmonary diseases [67].

The use of biodegradable materials may reduce potential toxicity; however, their physicochemical characteristics still need to be systematically evaluated before clinical application, as not all biomaterials are inherently safe for human use. The size of nanoparticles is critical for biodistribution, cell uptake and penetration of biological barriers, as smaller particles have increased potency, longer circulation time and reduced accumulation in the liver and spleen. The toxicity profile also varies among different classes of nanomaterials. Metallic nanoparticles may induce oxidative stress and inflammatory responses due to ion release and long-term accumulation, whereas ceramic nanomaterials generally exhibit improved biocompatibility but may influence cellular behavior depending on their composition and degradation products. Carbon-based nanomaterials possess unique mechanical and physicochemical properties; however, concerns regarding persistence, biodistribution, and long-term biological interactions remain under investigation [5,11,67,68]. Nevertheless, very small nanoparticles have an increased risk of diffuse or unpredictable toxicity in non-target tissues [5,68]. The shape also affects their in vivo behavior, with spherical nanoparticles being more efficient for drug transport, while they seem to be more often associated with non-specific filtration. In contrast, asymmetrical shapes, such as rod-like, disc-like, or lamellar structures, often exhibit prolonged circulation, better extravasation and more controlled biodistribution, thus reducing undesirable systemic accumulation. The development of “smart” nanoparticles with the ability to change shape and adjustable stiffness is of interest. Such an approach could find applications in periodontal regeneration by achieving increased local drug accumulation, stronger mechanical support scaffolds and at the same time reducing systemic toxicity in the blood, liver, kidneys and heart [11]. In the context of 3D-bioprinted scaffolds, the safety of nanomaterials is closely associated with the biocompatibility of biomaterials and their ability to mimic the natural properties of human tissues while minimizing immune responses and tissue degeneration. Despite the promising regenerative potential of multifunctional nanocomposite scaffolds, important biosafety and translational concerns still remain [5,67,68,69]. Furthermore, evidence from nanomedicine research suggests that only a limited number of nanoparticle-based systems successfully achieve clinical translation despite encouraging preclinical outcomes, mainly due to challenges related to biodistribution, targeting efficiency, reproducibility, large-scale manufacturing, and insufficient long-term safety evaluation [69,70]. Consequently, long-term monitoring and comprehensive toxicity assessment remain essential prerequisites for ensuring biomedical safety, preventing unforeseen adverse reactions, and supporting the safe clinical translation of bioprinting technologies for periodontal tissue engineering [5,19,70].

Overall, the clinical translation of multifunctional nanocomposite scaffolds requires a careful balance between regenerative efficacy and long-term safety [5,11,67,68]. Nonetheless, these systems offer significant advantages in terms of osteogenesis, angiogenesis, antibacterial activity, and controlled drug delivery; potential risks related to biodistribution, immunogenicity, and chronic toxicity must be thoroughly evaluated before routine clinical application [5,11,67,68,69]. Therefore, standardized safety assessment protocols and long-term clinical studies remain essential for their successful translation into clinical practice [19,68,69,70].

## 6. Current Challenges in Clinical Translation

The main challenge lies in the precise reproduction of the complex microstructure of the periodontal complex, which is organized on multiple levels and simultaneously requires the creation of tissues with different and often conflicting mechanical properties within the same scaffold. Despite advances in nanotechnology and 3D bioprinting, controlling scaffold architecture at the micro- and nanoscale, as well as accurately mimicking the mechanical properties of native tissues, remains technically challenging. Furthermore, a significant difficulty is understanding and controlling how cells respond to stimuli from their environment. The nanostructured features of the scaffolds, such as fiber orientation and nano-grooves, affect cell adhesion and orientation, while cells simultaneously receive biochemical and mechanical signals. The synchronized and controlled combination of these signals at the molecular level remains particularly challenging, as inadequate distribution may lead to conflicting stimuli and reduced regulation of cellular differentiation and tissue organization [71]. Another critical issue is the control of scaffold degradation rates, since premature degradation may result in loss of structural stability and undesirable tissue infiltration, thereby disrupting the coordinated regenerative process [52]. Further research remains essential to achieve adequate vascularization in thicker multilayered scaffolds, as the limited supply of oxygen and nutrients can negatively affect cell viability [71]. The regeneration of CM in vivo is particularly demanding, as it requires strict control of cellular differentiation and tissue maturation. Growth factors such as BMPs have so far shown regenerative potential, but their application is associated with supraphysiological doses, which increases cost and the risk of complications [16]. Multiphase scaffolds show encouraging results at the preclinical level; however, their high cost, increased fabrication time and limited reproducibility hinder their widespread clinical application [59]. At the same time, maintaining cell viability before and after bioprinting is critical, as the process can cause mechanical and physicochemical stress. Additionally, the selection of appropriate materials is complex, as scaffolds must combine low immunogenicity, good interaction with the host, sufficient mechanical support, and a controlled degradation rate without requiring surgical removal [61]. The transition to clinical practice is accompanied by significant ethical and regulatory issues due to the use of human cells and the lack of a comprehensive regulatory framework, despite existing guidelines [22,72]. Most currently available evidence originates from in vitro studies and animal models, whereas well-designed human clinical studies remain comparatively scarce. Recent systematic reviews and meta-analyses have demonstrated encouraging clinical outcomes following the use of stem cell-based regenerative approaches and advanced biomaterials, including improvements in clinical attachment level gain, probing depth reduction, and radiographic tissue regeneration [73]. Nevertheless, substantial heterogeneity in study design, biomaterial composition, scaffold architecture, outcome assessment, and follow-up duration continues to limit direct comparison between studies and restrict widespread clinical implementation [11,74]. Furthermore, long-term safety data regarding biodistribution, immunogenicity, biodegradation behavior, and chronic toxicity of nanomaterial-enhanced scaffold systems remain insufficiently established, highlighting the need for standardized translational protocols and larger clinical trials before routine clinical application can be achieved [5,11,69,74].

When compared with guided tissue regeneration (GTR), which remains the current gold standard for periodontal regeneration, multifunctional bioprinted nanocomposite scaffolds offer the potential for more precise and tissue-specific regeneration [6,7]. Their ability to combine spatial compartmentalization, controlled delivery of bioactive molecules, and patient-specific architectural design may help address some of the limitations associated with conventional regenerative approaches [2,4,62,66]. However, despite these promising features, most bioprinted scaffold systems remain at the preclinical stage, and further studies are required to establish their long-term safety, reproducibility, and clinical effectiveness [11,59,69,74].

The challenges and therapeutic approaches to periodontal tissue regeneration using 3D-bioprinted scaffolds are summarized in Table 3 below.

## 7. Emerging Trends and Future Directions

In recent years, 4D bioprinting and smart scaffolds have been steadily gaining ground in tissue regeneration [71,75]. Unlike 3D bioprinting, 4D (4-dimensional) introduces the time dimension, allowing 3D structures to dynamically change in response to stimuli such as temperature, pH or humidity [52]. Thus, scaffolds can adapt to the microenvironment and gradually degrade as new tissue forms [72]. Smart scaffolds mimic the natural extracellular matrix and actively interact with cells, regulating their behavior and enhancing the regeneration process [4]. Shape memory polymers (SMPs), which can automatically adapt to the defect, are of particular interest [71]. Although the potential of these technologies is significant and already recognized, more preclinical and clinical studies are required to evaluate their safety, performance and long-term stability, as well as to facilitate their transition from the laboratory to clinical practice [52,76,77].

A rapidly growing field in tissue engineering is bioprinting with the aid of artificial intelligence (AI), which utilizes machine learning and deep learning algorithms to analyze large datasets and recognize patterns. Through this approach, the design of scaffolds, biomaterials and printing parameters is optimized, reducing the need for extensive experimental testing and accelerating the development process. AI also contributes to predicting printability, improving mechanical properties and dimensional accuracy of scaffolds, while enhancing quality control through image analysis and detection of structural defects [78]. At the same time, it allows the adjustment of critical parameters, such as pressure, printing speed and layer thickness, improving cell viability. In periodontal regeneration, machine learning supports the development of personalized therapeutic approaches, leveraging data from imaging techniques and genetic information to design patient-specific scaffolds [72]. Overall, the integration of AI in bioprinting is expected to facilitate the transition from the laboratory to clinical practice, reducing costs and improving the effectiveness of bioprinted scaffolds [78].

Based on recent data, organ-on-a-chip systems represent a particularly promising approach for periodontal regeneration, primarily as preclinical study models rather than implantable scaffolds. They essentially function as an intermediate stage between in vitro and in vivo studies, allowing the reproduction of complex interfaces, such as those between hard and soft periodontal tissues, under dynamic flow and mechanical loading conditions [79,80]. Through these systems, it becomes possible to study biological interactions, osteogenesis, angiogenesis, and cellular communication in environments that approximate the natural microarchitecture of tissues, while simultaneously reducing the need for experimental animals and the risk of failure in subsequent stages [75]. At the same time, they have particular value in personalized medicine, as cells from the patient themselves can be used to test different therapeutic regimens before selecting the most appropriate one to be administered to the patient, in order to achieve a reduction in side effects and an increase in success rates [81]. Despite recent advances, their applications in periodontology remain limited and do not fully capture the complexity of the periodontal structures, making the development of more complex multicellular models necessary. At the same time, these systems are considered particularly useful platforms for supporting personalized approaches, utilizing cells from the patient themselves before transitioning to clinical practice [80,81]. A characteristic example is the “PDL-on-chip” system, which simulates the vascularized PDL and allows the study of its pathophysiology under in vivo conditions. It can reproduce inflammatory responses, functioning as a platform for testing regenerative strategies rather than as a therapeutic solution on its own [82].

Collectively, these emerging technologies have the potential to transform periodontal regeneration from a predominantly structural approach into a dynamic and personalized therapeutic strategy. Nevertheless, several challenges remain to be addressed, including manufacturing reproducibility, large-scale production, cost-effectiveness, long-term biosafety, and regulatory approval. Future progress will likely depend on the successful integration of advanced biomaterials, intelligent design tools, patient-specific treatment planning, and standardized translational frameworks capable of supporting routine clinical implementation [11,22,72].

## 8. Conclusions

The field of 3D scaffolds is constantly evolving and has paved the way for the simultaneous regeneration of AB, PDL and CM as they attempt to mimic the composition, spatial organization and architecture of the periodontal unit [4,52]. It also opens up opportunities for the construction of customized 3D scaffolds that are tailored to the specific needs of the patient, allowing for precise control of the shape and size of the regenerated tissues. Crucial to the success of 3D bioprinting in periodontal regeneration is the reinforcement of scaffolds with nanocomposite biomaterials and bioinks. Through the development of specialized nanomaterials, it is expected that holistic periodontal regeneration will be achieved, as long as these materials are biodegradable, biocompatible and non-toxic. These nanomaterials provide improved mechanical properties and the ability to respond to various stimuli, while also being able to be used as drug delivery nanosystems, providing a controllable structure, personalized surfaces and bioadhesive behavior [83]. In addition to nanoparticles, bioactive or growth factors can be incorporated into scaffolds, allowing for the regulation of the microenvironment, angiogenesis, and immunoregulation, which is supportive of overall regeneration [5]. Available preclinical evidence suggests that properly designed multiphasic scaffolds incorporating appropriate cells, nanomaterials, and bioactive molecules can support coordinated regeneration of AB, PDL, and CM, representing one of the most promising approaches in periodontal tissue engineering [60,84]. There are additional preclinical in vitro and in vivo studies that have demonstrated the simultaneous regeneration of the AB, PDL, and CM [59]. Nevertheless, most currently available multifunctional nanocomposite scaffold systems remain at the preclinical stage, with evidence derived predominantly from in vitro experiments and animal studies [11,59,74]. Nonetheless, several studies have reported encouraging regenerative outcomes; only a limited number of approaches have progressed to human clinical evaluation [77]. Furthermore, no clinically established scaffold platform capable of predictably regenerating the entire AB –PDL-CM complex has yet emerged [7,57,59]. Challenges related to manufacturing reproducibility, long-term biosafety, regulatory approval, and cost-effective large-scale production continue to hinder clinical translation. Therefore, substantial scientific, technological, and regulatory barriers must still be overcome before multifunctional bioprinted nanocomposite scaffolds can become routine clinical therapies [11,59,70].

## Figures and Tables

**Table 1:** Evaluation and comparison of the main 3D bioprinting techniques for periodontal regeneration (AB, PDL, CM).

Evaluation Criteria	Vat Polymerization	Extrusion Bioprinting	Droplet-Based Bioprinting	Laser Assisted Bioprinting
**Cell viability**	Affected by photoinitiators and UV exposure [20]	Depends on shear stress, bioink composition and printing parameters [17,18,20]	Generally maintained under optimized printing conditions [18]	Preserved due to nozzle-free deposition [14,17,28]
**Printing** **resolution**	Producing highly detailed structures [17,20,28]	Influenced by nozzle diameter and extrusion parameters [18,20,21]	Precise droplet deposition [13,17,18]	Precise cell and material placement [13,18,28]
**Mechanical strength**	Depend on resin formulation and crosslinking density [29]	Influenced by bioink viscosity and scaffold composition [20]	Often require post-processing to improve structural stability [23]	Depend on material composition and post-processing [28]
**Material** **viscosity**	Requires photocurable materials within a defined viscosity range [29]	Compatible with a broad viscosity range (≈30 mPa·s to >10^7^ mPa·s) [17,21]	Requires low-viscosity bioinks (≈3.5–12 mPa·s) [17,18,20,23]	Compatible with a relatively broad viscosity range (≈1–300 mPa·s) [13,17]
**Cell density**	Supports moderate cell loading [21]	Supports dense cell-laden bioinks [21]	More suitable for lower cell concentrations [21]	Supports moderate cell loading [21]
**Structural complexity**	Suitable for fabrication of complex geometries [29]	Structural complexity may be limited by printing fidelity and bioink properties [17]	Generate heterogeneous structures with controlled deposition [23]	Suitable for fabrication of complex architectures [28]
**Multi-material capability**	Limited compatibility with simultaneous multi-material fabrication [13,17]	Supports multi-material and multiphasic scaffold fabrication [17,18,20]	Multiple materials can be printed simultaneously [23]	Limited availability of compatible printable materials [29]
**Suitability for periodontal** **regeneration**	Precise fabrication of mineralized periodontal components [13,14,15,16]	Fabrication of cell-laden and multiphasic AB–PDL–CM scaffolds [18]	Applications requiring controlled material deposition and spatial organization [13,15,18,23]	Precise cell placement and fabrication of complex periodontal architectures [28]
**Cost**	Moderate equipment and material costs [14,17]	Relatively cost-effective compared with other bioprinting approaches [13,17,18]	Lower equipment costs but limited bioink compatibility [13,23]	Requires specialized and relatively expensive equipment [17,18]

**Table 2:** Representative preclinical and translational studies on nanocomposite and multiphasic scaffold systems for periodontal regeneration.

Scaffold Composition	Fabrication Strategy	Model	Main Outcomes	Key Limitations	Ref.
PLGA/PCL + AgNPs + pDA + collagen	Nanofibrous scaffold	Mouse model	~31.8% bone regeneration	Preclinical study	[46]
HA nanoparticles + hydrogel + PDLSCs	Hydrogel scaffold	Experimental model	Improved differentiation and stability	Limited translational data	[51]
PCL/collagen/cellulose acetate + ZIF-8-curcumin	Electrospinning + hydrogel	In vitro	Anti-inflammatory and regenerative effects	No in vivo validation	[54]
Layered membranes	Layer-by-layer engineering	Animal model	Periodontal restoration	Animal study	[65]
PCL/HA triphasic scaffold + amelogenin + CTGF + BMP-2	3D printing	In vivo and in vitro	Regeneration of AB, PDL and CM-like tissues	Preclinical stage	[66]
Chitin-PLGA + nanoactive glass ceramic + CEMP1 + PRP + FGF-2	Triple-layer scaffold	Animal model	Cell-free periodontal regeneration	No human validation	[4]

**Table 3:** Overview of challenges and emerging approaches in periodontal regeneration with 3D-bioprinted scaffolds.

Challenges	Description	Therapeutic Approaches	Reference
Complexity of periodontal tissues	Difficulty in simultaneous regeneration of AB, PDL, and CM with proper orientation	Multifunctional and multiphasic scaffolds, combination of bioprinting techniques	[1,6,59,71]
Limited vascularization	Insufficient blood supply to the scaffolds in large defects leads to low cell survival	Incorporation of angiogenic factors, use of stem cells, pre-vascularized scaffolds	[29,64,71]
Low cell viability	Mechanical/thermal stresses during printing affect cells	Development of mild techniques, optimization of biomaterials and rheological properties	[17,22,26,62]
Biocompatibility and safety	Potential toxicity or uncontrolled degradation of materials	Biodegradable and nanocomposite materials with controlled degradation	[11,19]
Chronic inflammation and microbial load affect regeneration	Chronic inflammation and biofilm hinder healing	Anti-inflammatory nanomaterials with antimicrobial action and targeted drug release	[6,40,47]
Clinical translation	High cost, lack of standardization, regulatory barriers	Standardization, automation, regulatory frameworks, personalized protocols	[19,22,59,72]

## Data Availability

Not applicable.

## Abbreviations

The following abbreviations are used in this manuscript:

AB	Alveolar Bone
PDL	PerioDontal Ligament
CM	Cementum
3D	3-dimensional
GTR	Guided Tissue Regeneration
TE	Tissue Engineering
ECM	ExtraCellular Matrix
CAD	Computer-Aided Design
SLA	Stereolithography
DLP	Digital Light Processing
cDLP	continuous Digital Light Processing
2PP	two-Photon Polymerization
PEG	PolyEthylene Glycol
PCL	PolyCaproLactone
CIJ	Continuous InkJet
DOD	Drop-On-Demand
AM	Additive Manufacturing
LBB	Laser-Based Bioprinting
LIFT	Laser-Induced Forward Transfer
EHD	ElectroHydroDynamic
UV	UltraViolet
dECM	decellularized ExtraCellular Matrix
RGD	Arginine-Glycine-Aspartate (Arg-Gly-Asp)
HA	Hydroxyapatite
β-TCP	β-Tricalcium Phosphate
α-TCP	α-Tricalcium Phosphate
BCP	Biphasic Calcium Phosphate (mixture of HA and TCP)
TTCP	Tetracalcium Phosphate
DCP	Brushite (Dicalcium Phosphate
MCPM	Monocalcium Phosphate Monohydrate
45S5 bioglass	Calcium sodium phosphosilicate
PMNC	Polymer Matrix Nanocomposites
CMNC	Ceramic Matrix Nanocomposites
MMNC	Metal Matrix Nanocomposites
PLGA	poly(lactic-co-glycolic acid)
nHA	nano-hydroxyapatite
pDA	polyDopamine
AuNPs	Gold nanoparticles
BMP-2	Bone Morphogenetic Protein 2
BGs	Bioactive Glasses
GelMA	Gelatin Methacrylate
CNTs	Carbon nanotubes
CNFs	Carbon nanofibers
DNA	Deoxyribonucleic acid
PDACS	polydopamine
hPDLSCs	human periodontal ligament stem cells
ALP	Alkaline Phosphatase
PDGF-BB	Platelet-Derived Growth Factor BB
CA	Cellulose Acetate
CEMP1	Cementum Protein 1
CGF	Cementum-derived Growth Factor
CAP	Cementum Attachment Protein
PGA	Poly(glycolic acid) (or Polyglycolide)
PDGF	Platelet-Derived Growth Factor
LBL	Layer-by-Layer
CTGF	Connective Tissue Growth Factor
PRP	Platelet-Rich Plasma
FGF-2	Fibroblast growth factor 2
ROS	Reactive Oxygen Species
4D	4-dimensional
SMPs	Shape memory polymers
AI	Artificial Intelligence
